# UNC13C Suppress Tumor Progression via Inhibiting EMT Pathway and Improves Survival in Oral Squamous Cell Carcinoma

**DOI:** 10.3389/fonc.2019.00728

**Published:** 2019-08-08

**Authors:** Bharath Kumar Velmurugan, Kun-Tu Yeh, Ming-Ju Hsieh, Chung-Min Yeh, Chia-Chieh Lin, Chuan-Yu Kao, Lan-Ru Huang, Shu-Hui Lin

**Affiliations:** ^1^Toxicology and Biomedicine Research Group, Faculty of Applied Sciences, Ton Duc Thang University, Ho Chi Minh City, Vietnam; ^2^Department of Pathology, Changhua Christian Hospital, Changhua City, Taiwan; ^3^School of Medicine, Chung Shan Medical University, Taichung City, Taiwan; ^4^Institute of Medicine, Chung Shan Medical University, Taichung City, Taiwan; ^5^Department of Holistic Wellness, Mingdao University, Changhua City, Taiwan; ^6^Oral Cancer Research Center, Changhua Christian Hospital, Changhua City, Taiwan; ^7^Graduate Institute of Biomedical Sciences, China Medical University, Taichung City, Taiwan; ^8^Department of Medical Technology, Jen-Teh Junior College of Medicine, Nursing and Management, Miaoli City, Taiwan; ^9^Department of Medical Laboratory Science and Biotechnology, Central Taiwan University of Science and Technology, Taichung City, Taiwan

**Keywords:** UNC13C, oral cancer, overall survival rate, EMT, Vimentin, Claudin1

## Abstract

Potential function of UNC13C in variety of cancers including, oral squamous cell carcinoma (OSCC) remains obscure. In the present study, immunohistochemical staining in tissue microarrays containing 268 OSCC samples showed that UNC13C protein levels were inversely correlated with AJCC Stage III and IV (*P* = 0.002) and death (*P* = 0.0134). Patients with lower UNC13C expression had a significantly shorter survival (*P* = 0.0231) than those with higher UNC13C expression. We also identified decreased overall UNC13C expression in oral cancer cell lines. In addition, our functional analysis of UNC13C shows that overexpression of UNC13C inhibited migration and invasion capacities of SCC-9 and SAS cells compared with the empty plasmid transfected cells. Further experiments suggested that transcription factors (Slug, Snail, Twist, and ZEB1) and mesenchymal marker (Vimentin) were down regulated and Tight Junction Protein (Claudin1) was up regulated after UNC13C overexpression in SCC9 and SAS cells. The novel role of UNC13C is revealed for the first time in OSCC. In summary, these results suggest that UNC13C as a novel tumor suppressor and an essential regulator of EMT signaling pathway during OSCC progression, and thus it could be used as a target for preventing oral cancer metastasis.

## Introduction

Oral squamous cell carcinoma (OSCC) is one of the most common cause of cancer death in the worldwide (1), particularly in Taiwan (2). OSCC is a prevalent malignancy that represents the fourth most common cancer affecting males (M:F = 10.8:1; 6,308:582 new cases in 2012, per the Taiwan Cancer Registry; http://tcr.cph.ntu.edu.tw/main.php?Page=A1). Despite of the improved diagnostic techniques and treatment methodology, survival rate of oral cancer patients has not improved (3) because most of OSCC cases were detected at advanced stage, the main reasons is due to lack of early molecular marker. Early detection is one of the effective ways to improve patient survival. Therefore, it is important to screen better biomarkers to improve the detection efficiency in oral cancer patients.

UNC13C or Munc13-3 plays a role in vesicle maturation during exocytosis and in neurotransmitter release. Previous study has found that, UNC13C was frequently altered (10–22% of patients) in Gingivo-buccal oral squamous cell carcinoma (OSCC-GB) patients (4). UNC13C is predicted in regulating neurotransmitter release (5); alterations in UNC13C could be associated to predisposition to tobacco addiction, which might enhance the risk of oral cancer. However, the function of UNC13C in tumor initiation and progression is unclear. Moreover, there are currently no reports describing the role of UNC13C in OSCC.

In this study, we showed that UNC13C expression in OSCC tissues was significantly down-regulated compared with surrounding normal marginal tissues and reduced UNC13C expression predicted poor survival rate in OSCC patients. *In vitro* experiments confirmed the inhibitory effects of UNC13C on cell migration and invasion. Additionally, we investigated the possible roles and molecular mechanisms of UNC13C in oral cancer cells using *in vitro* models. Our study provides novel insights into the tumor suppressive role of UNC13C in OSCC, restoring UNC13C expression may be a novel treatment strategy for OSCC.

## Results

### UNC13C Down-Regulated in Human OSCC Tissues

**Figure 1:**
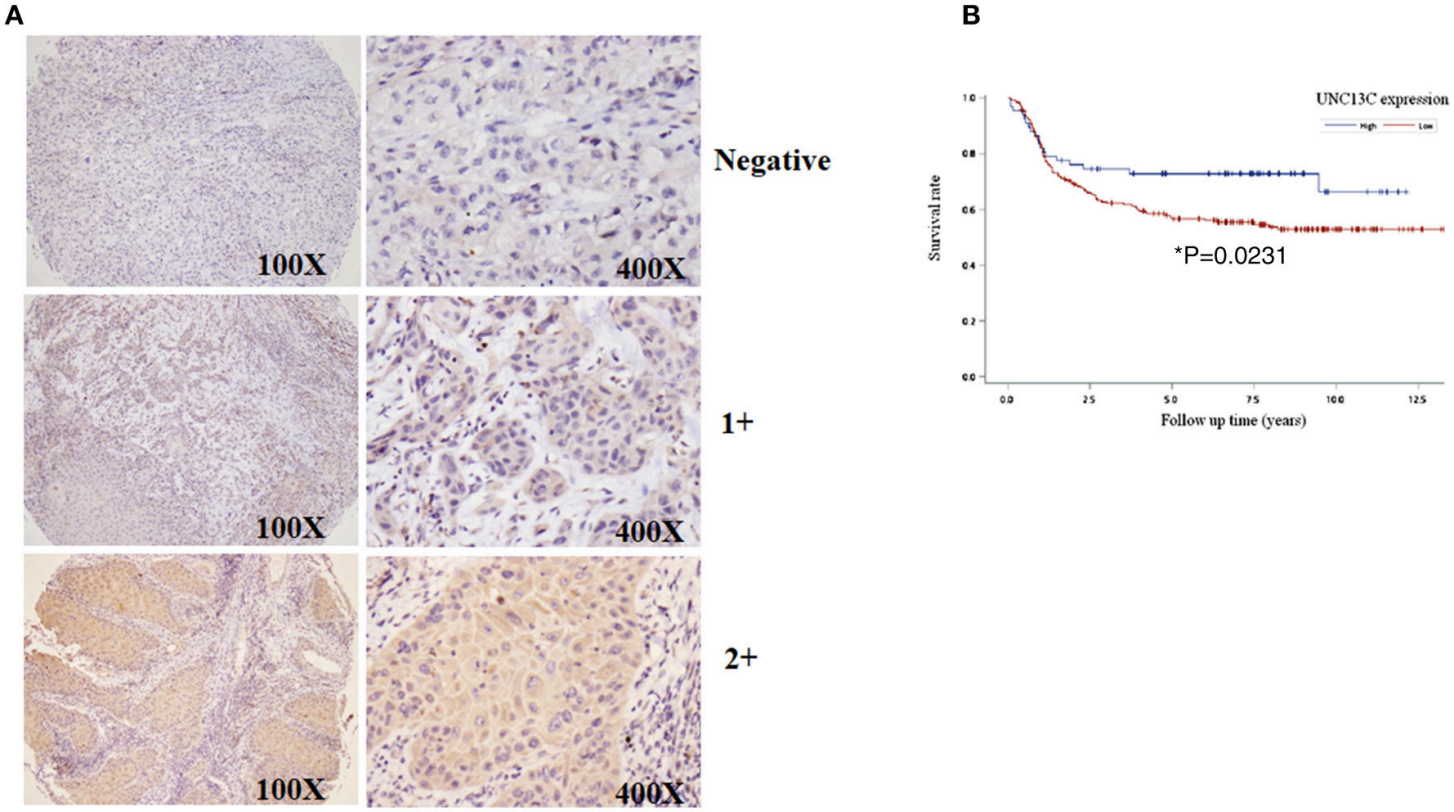
Downregulation of UNC13C in OSCC tissues. **(A)** The protein levels of UNC13C were tested by immunohistochemistry staining. **(B)** Kaplan–Meier analysis of UNC13C expression in patients with OSCC. Low expression level of UNC13C was associated with worse overall survival of OSCC patients (^*^P = 0.0231).

To determine the role of UNC13C in oral cancer, the expression level of UNC13C was first examined in oral cancer tissues (*n* = 268) using Immunohistochemistry staining. The patients' characteristics, including their sex, age, tumor stage, lymph node status, AJCC cancer stage, and histological grade are listed in Table 1. UNC13C expression was evaluated and scored by two pathologists, tumor tissues showed positive and negative staining of UNC13C (Figure 1A). Of the OSCC specimens examined, low UNC13C expression was detected in 190 of the 257 (74.0%) patients and high UNC13C expression was detected in 67 of the 257 (26.0%) patients.

**Table 1:** Demographic and characteristics of oral cancer patients.

**Factors**	**Number**	**%**
**Gender**		
Female	15	5.6
Male	253	94.4
**Age, year**		
≤49	83	31.0
50–59	95	35.5
60–69	59	22.0
≥70	31	11.6
**T (Tumor size)**		
I	68	25.4
II	84	31.3
III	21	7.8
IV	95	35.5
**N (Lymph node)**		
N0	173	64.6
N1	33	12.3
N2	59	22.0
N3	3	1.1
**M (Metastasis)**		
No	267	99.6
Yes	1	0.4
**AJCC Cancer stage**		
I	53	19.8
II	56	20.9
III	31	11.6
IV	128	47.8
**Histological grade**		
Well	42	15.7
Moderate	218	81.3
Poor	8	3.0

### Association of UNC13C Expression With Clinico-Pathological Parameters

We then analyzed the relationship between UNC13C expression and clinicopathological variables in OSCC (Table 2). UNC13C expression was significantly correlated with AJCC stage (*P* = 0.002) and death (*P* = 0.0134) (Table 2). However, UNC13C expression was not associated with the other clinicopathological features, such as TNM stage and histologic grade, was observed (*P* > 0.05) in our study. Survival analysis showed that patients with lower UNC13C expression had a shorter survival time than those with higher UNC13C expression (Figure 1B, *P* < 0.05).

**Table 2:** Clinicopathologic factors associated with UNC13C expression.

	**UNC13C**			
**Factors**	**Low no. (*n* = 190)**	**High no. (*n* = 67)**	^*^aOR (95% CI)^**a**^	***P-value***
Tumor size (SD)	3.06 (1.62)	2.93 (1.53)		
**T Classification**				
I/II	102	43	1	
III/IV	88	24	0.65 (0.36–1.15)	0.3638
**N (Lymph node)**				
N0/N1	146	54	1	
N2/N3	44	13	0.79 (0.36–1.60)	0.4
**M (Metastasis)**				
No	189	67	1	
Yes	1	0	ND	
**AJCC Cancer stage**				
Early stage (I/II)	71	36	1	
Advance stage (III/IV)	119	31	0.51 (0.29–0.86)	0.002
**Histological grade**				
Well	31	10	1	0.184
Moderate/poor	159	57	1.09 (0.49–2.49)	
**Death**				
No	104	48	1	
Yes	86	19	0.47 (0.25–0.85)	0.0134

a *Adjusted odds ratio (aOR) was controlled for gender and age*.

* *P < 0.05; ND, not determined*.

Furthermore, we performed multivariate Cox regression analysis to examine whether UNC13C expression was an independent prognostic factor for OSCC patients Table 3. After adjustment with age and gender, we observed that T stage (aHR, 2.1; 95% CI, 1.4–3.0; *P* = 0.0001), N stage (aHR, 3.1; 95% CI, 2.1–4.6; *P* < 0.0001), AJCC tumor stage (aHR, 2.7; 95% CI, 1.7–4.3; *P* < 0.0001), Tumor differentiation (aHR, 2.9; 95% CI, 1.4–6.1; *P* = 0.0037) and UNC13C expression (aHR, 0.59; 95% CI, 0.35–0.95; *P* = 0.037) were independent prognostic factors for OSCC.

**Table 3:** The effect of clinicopathologic factor and UNC13C expression on mortality density and adjusted hazard ratio (aHR) among OSCC patients.

**Factors**	**No. of patient**	**Follow-up (person-year)**	**No. of death**	**Mortality density^**a**^**	**aHR^**b**^**	**(95% CI)**	**Interaction *P*-value**
**Overall Mortality From Primary Malignancy To Death**							
**T classification**							
I/II	152	905.32	52	5.7	1		
III/IV	116	498.87	65	13.0	2.1	(1.4–3.0)	0.0001
**N classification**							
N0/N1	206	1248.94	73	5.8	1		
N2/N3	62	155.25	44	28.3	3.1	(2.1–4.6)	<0.0001
**AJCC tumor stage**							
I/II	109	731.57	30	4.1	1		
III/IV	159	672.62	87	12.9	2.7	(1.7–4.3)	<0.0001
**Tumor differentiation**							
Well	42	277.29	9	3.2	1		
Moderate/poor	226	1126.89	108	9.6	2.9	(1.4–6.1)	0.0037
**UNC13C expression**							
Low	190	1025.45	83	8.1	1		
High	67	349.61	19	5.4	0.59	(0.35–0.95)	0.0377

a *Mortality density was displayed as per 100 people-years*.

b *aHR was adjusted for gender and age*.

### UNC13C Influenced Migration and Invasion of Oral Cancer Cells

**Figure 2:**
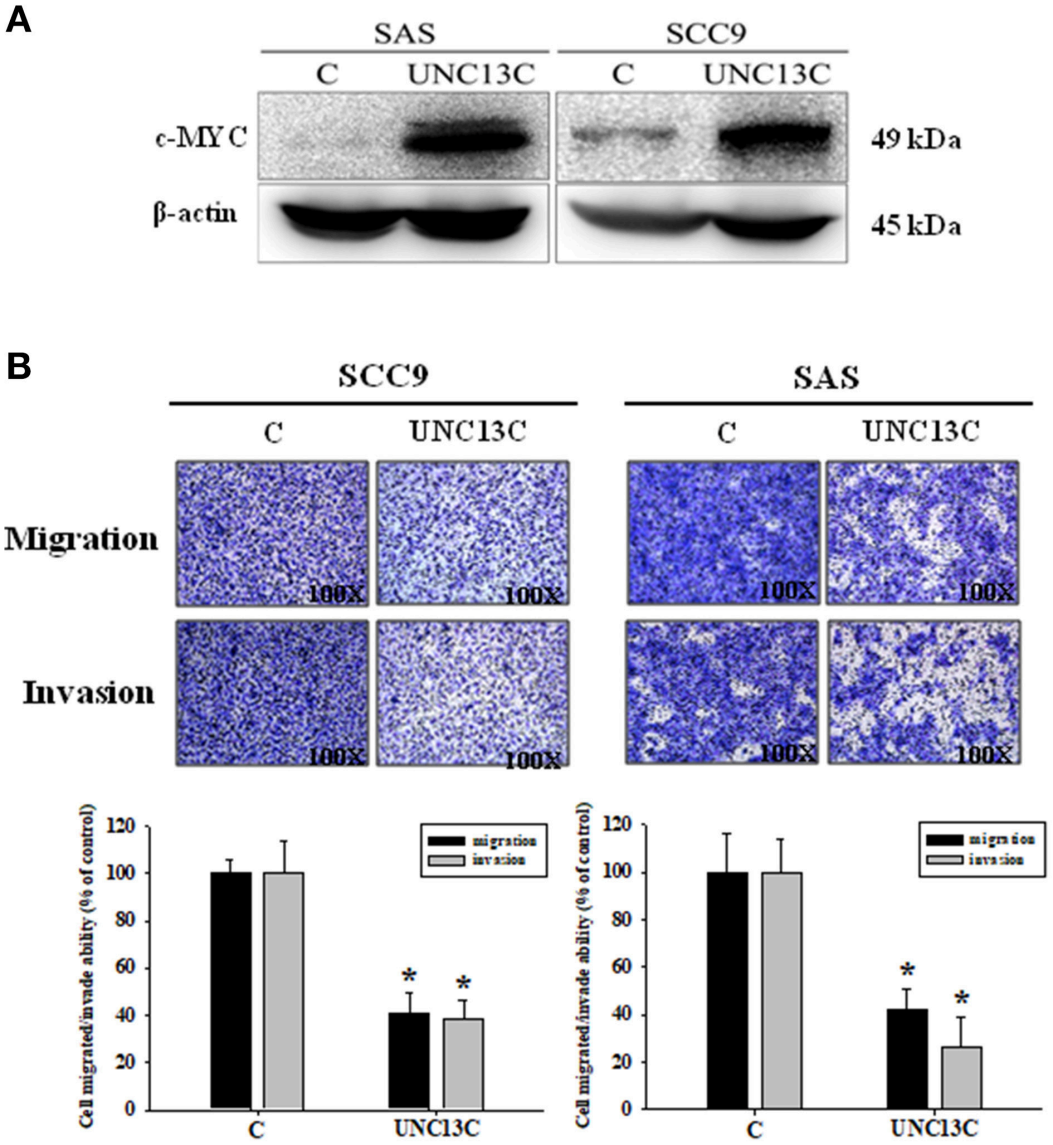
Overexpressing UNC13C inhibits the migration and invasion of OSCC cells. SCC-9 and SAS cells were transfected with pUNC13C-Myc and then analyzed for **(A)** c-MYC protein expression using western blotting. **(B)** The migration and invasion capacities were determined by Transwell assay. Bar graphs represent quantitative data from three independent experiments. ^*^P < 0.05 vs. non-transfected cells.

Firstly, we measured the expression of UNC13C in different OSCC cell lines, and the results showed that UNC13C was not expressed in OSCC cell lines (Data not shown). To elucidate the functional relevance of UNC13C in OSCC progression, we performed functional analysis in SAS and SCC-9 cell lines after UNC13C expression. As shown in Figure 2A, transfection of the plasmid pUNC13C-Myc into SAS and SCC-9 cells led to a significant increase in endogenous UNC13C compared with empty plasmid transfected cells (Figure 2A).

In order to determine the role of UNC13C in metastasis of OSCC, we performed transwell assay to assess the impact of UNC13C expression on cell migration and invasion abilities of SCC9, SAS, and OSC-20 cells. Transwell assays revealed that the migration and invasion abilities of the OSCC cells were significantly impaired following upon UNC13C overexpression. As shown in Figure 2B, SCC-9 and SAS cell lines showed high metastasis and invasive ability.

**Figure 3:**
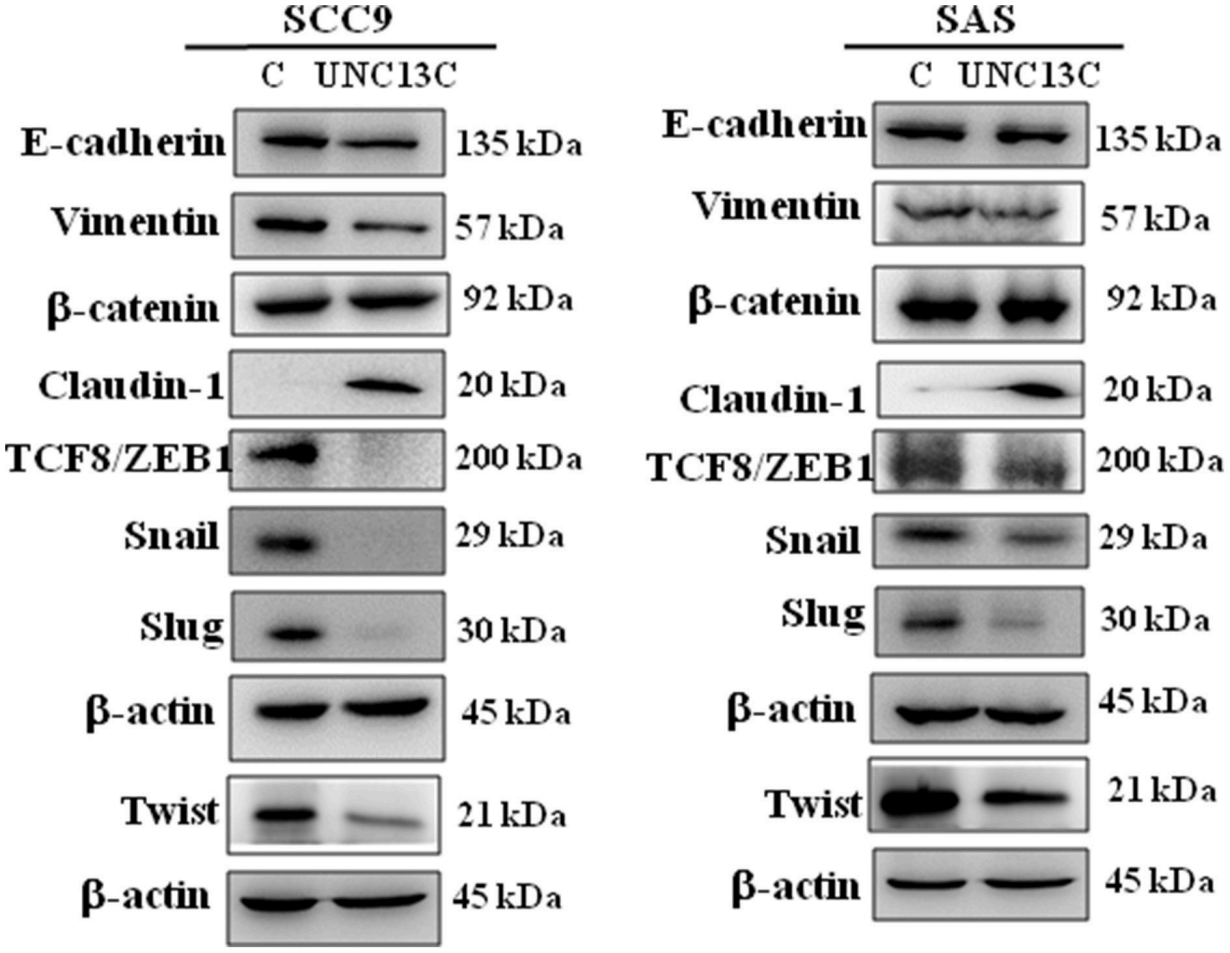
Expression levels of UNC13C affect EMT markers. SCC-9 and SAS oral cancer cells were transfected with pUNC13C-Myc plasmid and then analysed for EMT markers expression by Western blotting. All the experiments were repeated three times independently. β-actin was used as the loading control.

It is well-known that epithelial–mesenchymal transition (EMT) is an important event involved in the initiation of tumor invasion; the impact of UNC13C on the expression of EMT markers was analyzed in SCC-9 and SAS cells. UNC13C overexpression resulted in repression of E-cadherin, Vimentin, Twist, TCF8/ZEB1, Snail, and Slug, as well as induction of Claudin-1 expression in SCC-9 and SAS cells (Figure 3), while β-catenin exhibited no significant difference after UNC13C overexpression in SCC-9 and SAS cells. Taken together, these results demonstrate that the loss of UNC13C induces EMT and promotes migration, invasion of OSCC cells.

## Discussion

Here, we showed for the first time that UNC13C down regulation in OSCC tissues, consistently with these results, UNC13C was reduced in OSCC cell lines (Data not shown). In addition, UNC13C expression was significantly correlated with clinical stage, death, and overall survival of OSCC patients. Furthermore, multivariate analysis identified that UNC13C protein level may serve as an independent prognostic factor in patients with OSCC. For validating the role of UNC13C in EMT pathway *in vitro*, oral cancer cells analyzed for migration and invasion ability. As expected, impaired migration, and invasion were observed following UNC13C overexpression. All of these data indicate that UNC13C acts as a tumor suppressor in OSCC and that the downregulation of UNC13C may promote progression and metastasis of OSCC.

Metastasis is a multifactorial process (6), during the metastatic event epithelial cells lose their polarity, and acquire migratory and invasive abilities (7, 8). We observed that UNC13C overexpression in oral cancer cells down regulated cellular migration and invasion and undergo a morphologic change like EMT. Recently, several factors that regulate EMT have been identified, SNAIL (SNAI1), SLUG (SNAI2), TWIST1, ZEB1, and ZEB2 (9, 10). In correlation with these altered migration and invasive capacities, we found that UNC13C stimulation induced tight Junction Protein -Claudin-1 expression and inhibited mesenchymal marker—Vimentin and transcription factors -Slug, Snail, Twist, and ZEB1 in both SCC-9 and SAS cell lines. Whereas, in SAS cell line, UNC13C overexpression decreased Vimentin, ZEB1, Snail and Slug expression and increased tight junction proteins Claudin-1. Decreased E-cadherin expression plays an important factor in cancer metastasis (11), however in our study UNC13C expression was negatively associated with E-cadherin expression in both SCC9 and SAS cell lines. The importance of the EMT in UNC13C-related metastatic functions of oral cancer needs further research. These results prove that UNC13C could inhibit cell migration, and invasion of OSCC cells through Caludin-1/Snail/Slug/Twist/ZEB1/Vimentin-signaling pathway.

In conclusion, this is the first study to examine the expression of UNC13C in OSCC. UNC13C expression was down regulated in OSCC samples, which predicts poor survival in patients with OSCC. Detection of UNC13C down-regulation in OSCC might be used to identify patients with poor prognosis in the future. In addition, our results suggest that restoration of UNC13C function represents a potential therapeutic strategy for treatment of metastatic OSCC.

## Materials and Methods

### Reagents

Antibodies were purchased from Cell signaling Technology (Danvers, MA), UNC13C antibody (ab122725) was purchased from Abcam, Cambridge, UK), β-actin (MABT825) was obtained from Sigma-Aldrich (St. Louis, MO, USA) and c-Myc antibody (ab32072) was obtained from Abcam (Cambridge, UK), all antibodies were stored at −20°C.

### Participants and Clinical Tissues

To evaluate the association of UNC13C expression with clinical/pathological factors and patient survival, a total of 268 oral cancer tissues samples (from January 2000 to December 2008) were obtained from 253 male and 15 female patients (age ranged from 30 to 90 years old) in Changhua Christian Hospital. Staging was classified according to the sixth edition of TNM staging system of AJCC (American Joint Committee on Cancer). The main treatment was tumor removal and radical neck dissection, including post-operation irradiation as well as selective patients treated with 5-FU and cisplatin chemotherapy.

### Tissue Microarray and Immunohistochemical Staining

Tissue microarray and Immunohistochemical staining was performed as described previously (12). Then slides were incubated with UNC13C antibody (ab122725, Abcam, Cambridge, UK) in room temperature for 20 min. After washing three times with PBS, the sections were incubated with appropriate peroxidase-labeled secondary antibodies for 30 min at room temperature. The sections were washed three times with PBS and then labeled by diaminobenzidine and counterstained with Mayer's haematoxylin, dehydrated and mounted.

Two experienced pathologists (Kun-Tu Yeh and Chang Wei-Hsiang) independently assessed the results of immunohistochemical staining and a final agreement was obtained for each score at a discussion microscope. The UNC13C expression levels were divided to three groups, negative (−), low expression (1+) and high expression (2+).

### Cell Culture

SCC-9, the human tongue squamous carcinoma cell lines, was obtained from ATCC (Manassas, VA). The other cell lines SAS, were purchased from and validated by the Japanese Collection of Research Bioresources Cell Bank (JCRB, Shinjuku, Japan). SCC-9 cells were cultured in Dulbecco's Modified Eagle Medium (DEME; Life Technologies, Grand Island, NY) supplemented with Ham's F12 Nutrient Mixture (Life Technologies, Grand Island, NY), 10% fetal bovine serum (FBS), 1.2 g/L sodium bicarbonate, 15 mM HEPES, 1% penicillin/streptomycin, hydrocortisone (400 ng/ml), 1% nonessential amino acids (NEAA). SAS cells were cultured in DMEM/F12 (Life Technologies, Grand Island, NY) supplemented with 10% FBS, 1.2 g/L sodium bicarbonate, 15 mM HEPES, 1% penicillin/streptomycin. All cell cultures were maintained in 5% CO_2_ at 37°C.

### Cell Transfection

Prior transfection, cells were plated on 6-cm tissue culture dishes at a density of 4 × 10^5^ cells/dish for overnight. A mixture of 4 μg DNA of pUNC13C-Myc, a constructed UNC13C expression plasmid (OriGene), and 12 μl TurboFect reagent (Thermo Fisher Scientific) in 400 μl DMEM/F12 (serum free) was added to cells and incubated for 24 h.

### Cell Migration and Invasion Assays

Cell migration and invasion was assayed according to the methods described previously (13, 14). After transfection for 24 h, the SCC-9 and SAS cells were harvested and seeded to upper Transwell (Greiner Bio-One, North Carolina, USA), and DMEM-F12 medium supplemented with 10% FBS (600 μl) was added in advance into Transwell lower chambers in a 24-well plate, then incubated for 24 h at 37°C. The invasion assay was carried out as described above used matrigel (25 mg/50 mL, 60 μl; BD Biosciences, MA) coating on the upper Transwell. Finally, the migrating or invading cells were fixed with methanol and stained with Giemsa for 2 h, and used a light microscope to count the cells.

### Western Blot Analysis

The cell lysates preparation using the methods as previously described (15, 16). The cell extracts were separated by 8 or 10% polyacrylamide gel and transferred onto a polyvinylidene fluoride (PVDF) membrane. The membrane was blocked in TBS-T buffer (20 mM Tris, 137 mM NaCl, pH 7.4) with 5% nonfat milk for 1 h, and the blots were probed with the indicated primary antibodies for 24 h at 4°C. After washing steps with TBS-T (10 min each) for three times, the blots were incubated with horseradish peroxidase anti-mouse IgG or goat anti-rabbit for 1 h at room temperature. Last, the blots were visualized by ECL reagent (Millipore), and the protein expression was detected by chemiluminescence. The relative photographic density was quantified by ImageQuant LAS 4000 mini Biomolecular Imager (GE Healthcare Bio-Sciences AB, Björkgatan 30 751 84 Uppsala, Sweden). EMT antibodies for E-cadherin [(24E10) Rabbit mAb #3195], Vimentin [(D21H3) XP® Rabbit mAb #5741], TCF8/ZEB1 [(D80D3) Rabbit mAb #3396], Snail-1 [(Rabbit mAb #3879), Slug [(C19G7) Rabbit mAb #9585], Caludin-1 [(D5H1D) XP® Rabbit mAb #13255], β-catenin [(D10A8) XP^®^ Rabbit mAb #8480] were obtained from Cell Signaling; antibodies for EMT were obtained from Cell Signaling EMT antibody sampler kit #9782. Twist (GTX127310) antibody was obtained from GeneTex (Hsinchu City, Taiwan).

### Statistical Analysis

Results were expressed as the means ± standard deviation (SD) of at least three independent experiments. Statistical analysis was performed using version 9.4 of the SAS software package (SAS Institute, Inc.; Cary, NC). Data comparisons were used Student's *t*-test between control and treated groups, when *P* < 0.05 was considered statistically significant. The associations of clinicopathologic factor and UNC13C expression were examined by logistic regression with adjustment for age and gender.

Cox's proportional hazards regression model was utilized for multivariate analyses to screen independent risk factors for prognosis of OSCC patients. Survival outcomes were summarized by the Kaplan-Meier method. *P*-values were obtained from log-rank tests for the homogeneity of Kaplan-Meier curves between high and low UNC13C expression.

## Ethics Statement

This study was approved by the Ethics Committees of Changhua Christian Hospital (Changhua, Taiwan) to use decoded tissue samples, and we adhered to the guidelines approved by (CCH IRB Nos. 181210 and 170413).

## Author Contributions

BV, K-TY, and M-JH: study design, experimental works, and manuscript drafting. C-MY, C-CL, and C-YK: manuscript drafting and analysis. L-RH and S-HL: experimental work.

### Conflict of Interest Statement

The authors declare that the research was conducted in the absence of any commercial or financial relationships that could be construed as a potential conflict of interest. The reviewer C-CY declared a shared affiliation, with no collaboration, with K-TY and M-JH to the handling editor at the time of review.
